# A dual-center cohort study on the association between early deep sedation and clinical outcomes in mechanically ventilated patients during the COVID-19 pandemic: The COVID-SED study

**DOI:** 10.1186/s13054-022-04042-9

**Published:** 2022-06-15

**Authors:** Robert J. Stephens, Erin M. Evans, Michael J. Pajor, Ryan D. Pappal, Haley M. Egan, Max Wei, Hunter Hayes, Jason A. Morris, Nicholas Becker, Brian W. Roberts, Marin H. Kollef, Nicholas M. Mohr, Brian M. Fuller

**Affiliations:** 1 Department of Emergency Medicine, Washington University School of Medicine in St. Louis, Campus Box 8054, St. Louis, MO 63110 USA; 2 Division of Critical Care, Departments of Emergency Medicine and Anesthesia, Roy J. and Lucille A. Carver College of Medicine, University of Iowa, 200 Hawkins Drive, 1008 RCP, Iowa City, IA 52242 USA; 3 Washington University School of Medicine in St. Louis, St. Louis, MO 63110 USA; 4 Carver College of Medicine, University of Iowa Hospitals and Clinics, Iowa City, USA; 5 Department of Emergency Medicine, Harvard-Affiliated Emergency Medicine Residency, Mass General Brigham, Boston, MA 02115 USA; 6 Department of Emergency Medicine, Mount Sinai Morningside/West, New York, NY 10025 USA; 7 Department of Emergency Medicine, Cooper University Hospital, One Cooper Plaza, Camden, NJ K152 USA; 8 Division of Pulmonary and Critical Care Medicine, Department of Medicine, Washington University School of Medicine in St. Louis, St. Louis, MO 63110 USA; 9 Division of Critical Care, Departments of Anesthesiology and Emergency Medicine, Washington University School of Medicine in St. Louis, St. Louis, MO 63110 USA

**Keywords:** COVID, Deep sedation, Emergency department, Mechanical ventilation

## Abstract

**Background:**

Mechanically ventilated patients have experienced greater periods of prolonged deep sedation during the coronavirus disease (COVID-19) pandemic. Multiple studies from the pre-COVID era demonstrate that early deep sedation is associated with worse outcome. Despite this, there is a lack of data on sedation depth and its impact on outcome for mechanically ventilated patients during the COVID-19 pandemic. We sought to characterize the emergency department (ED) and intensive care unit (ICU) sedation practices during the COVID-19 pandemic, and to determine if early deep sedation was associated with worse clinical outcomes.

**Study design and methods:**

Dual-center, retrospective cohort study conducted over 6 months (March–August, 2020), involving consecutive, mechanically ventilated adults. All sedation-related data during the first 48 h were collected. Deep sedation was defined as Richmond Agitation-Sedation Scale of − 3 to − 5 or Riker Sedation-Agitation Scale of 1–3. To examine impact of early sedation depth on hospital mortality (primary outcome), we used a multivariable logistic regression model. Secondary outcomes included ventilator-, ICU-, and hospital-free days.

**Results:**

391 patients were studied, and 283 (72.4%) experienced early deep sedation. Deeply sedated patients received higher cumulative doses of fentanyl, propofol, midazolam, and ketamine when compared to light sedation. Deep sedation patients experienced fewer ventilator-, ICU-, and hospital-free days, and greater mortality (30.4% versus 11.1%) when compared to light sedation (*p* < 0.01 for all). After adjusting for confounders, early deep sedation remained significantly associated with higher mortality (adjusted OR 3.44; 95% CI 1.65–7.17; *p* < 0.01). These results were stable in the subgroup of patients with COVID-19.

**Conclusions:**

The management of sedation for mechanically ventilated patients in the ICU has changed during the COVID pandemic. Early deep sedation is common and independently associated with worse clinical outcomes. A protocol-driven approach to sedation, targeting light sedation as early as possible, should continue to remain the default approach.

**Supplementary Information:**

The online version contains supplementary material available at 10.1186/s13054-022-04042-9.

## Introduction

Approximately 95% of all critical care interventional trials have failed to demonstrate benefit on clinical outcomes [1]. Despite this, outcomes for the critically ill have improved over the last several decades, owing not to disease- or syndrome-specific interventions, but secondary to improved supportive routine care. Generated from well-designed clinical trials and now guideline-supported, some of these routine care practices include lung-protective ventilation with lower tidal volume, conservative fluid management, the use of checklists, and early mobility [2–5]. Sedation management is another critical supportive therapy in mechanically ventilated patients. Specifically, a protocol-driven approach, which favors paired spontaneous awakening (SAT) and breathing (SBT) trials, along with light levels of sedation, improves outcome [6–12]. The early period of respiratory failure [i.e., the emergency department (ED) and first 48 h of intensive care unit (ICU)] may be especially critical to reduce the overall time spent with periods of deep sedation and coma [13–19].

However, there is little rigorous data on sedation depth and its impact on outcome for mechanically ventilated patients during the coronavirus disease (COVID)-19 era. As an example, a PubMed search (conducted on October 7, 2021) for “COVID-19” yielded 184,897 results; “COVID-19 AND sedation” yielded only 287, of which only one cohort study examined the impact of sedation depth on outcome [20]. In a comparison of patients with COVID-19-associated acute respiratory distress syndrome (ARDS) with historical ARDS controls, deep sedation and coma were common and associated with increased mortality [20]. High rates of delirium and coma have been observed in critically ill patients with COVID-19 infection [21]. Concerns have been raised that surges of COVID-19 cases have impacted the care of critically ill patients without COVID-19 disease, potentially worsening outcomes [22]. Overall, these findings suggest that the impact of early deep sedation on outcome during the COVID-19 pandemic, for patients with and without COVID-19, is incompletely understood.

We therefore conducted the COVID-SED Study to: (1) further characterize ED and ICU sedation practices during the COVID-19 pandemic; and (2) test the hypothesis that early deep sedation is associated with worse clinical outcomes.

## Methods

### Study design

This is a retrospective cohort study conducted over 6 months (March–August, 2020), involving consecutive adult mechanically ventilated patients admitted to ICUs at Barnes-Jewish Hospital in Saint Louis and at the University of Iowa Hospital, both in the United States. Both sites use ICU sedation protocols, which advocate for addressing analgesia first and controlling pain, then addressing sedation. Sedation depth is monitored using a validated scale including the Richmond Agitation-Sedation Scale (RASS) and the Riker Sedation-Agitation Scale (SAS). Benzodiazepines are avoided if possible. For difficult to sedate patients, co-sedation with ketamine, antipsychotics, or other agents is included. Delirium is monitored with CAM-ICU at least every 12 h.

The study is reported in accordance with the Strengthening Reporting of Observational Studies in Epidemiology (STROBE) Statement. (Additional file 1) [23]. The Institutional Review Board (IRB) and Human Research Protection Office (HRPO) at each site approved the study with waiver of informed consent prior to study initiation (IRB # 202009119 and 202009604).

### Participants

All consecutive mechanically ventilated adult patients admitted to the ICU from the ED were screened via established electronic screening procedures. Inclusion criterion: (1) age ≥ 18 years; and (2) receipt of mechanical ventilation via an endotracheal tube. In addition to mechanically ventilated patients admitted from the ED, all other mechanically ventilated COVID-19 patients admitted to the intensive care unit were screened for inclusion. This was done to capture all patients with COVID-19 during the 6-month enrollment period, provided they satisfied all other inclusion and exclusion criteria. Exclusion criteria targeted patients in whom duration of mechanical ventilation was unlikely to be altered by sedation management or those in whom acute injury could act as a confounder with sedation depth: (1) death or transition to comfort measures within 24 h; (2) acute neurologic injury (e.g. stroke, intracranial hemorrhage, traumatic brain injury, cardiac arrest with residual neurologic deficit, status epilepticus, drug overdose, fulminant hepatic failure); (3) transfer to another hospital; (4) chronic/home ventilation; (5) direct admission to the operating room (OR) from the ED; and (6) extubation in the ED.

### Assessments and outcome measures

Clinical variables and outcome measures were objective to ensure ease of abstraction from the electronic medical record. Data were collected and entered into a database with Research Electronic Data Capture (REDCap) tools [24, 25]. Team members were trained regarding data abstraction. Data quality checks were performed with manual and automated methods, and by enforcing plausible data ranges in the REDCap fields. Prior to analysis, the database was screened for implausible values and the electronic medical record was used to recheck any flagged data.

Baseline data including age, gender, weight, race, comorbid medical conditions, COVID-19 status, vital signs, laboratory values, indication for mechanical ventilation, and ventilator settings were recorded. Process of care variables included ED length of stay, antibiotic use, and vasopressor use. Illness severity was assessed with the modified sequential organ failure assessment (SOFA) score [26, 27]. Indication for mechanical ventilation was obtained according to the notes in the electronic medical record and adjudicated by data abstractors.

Sedation-related data included induction agents and neuromuscular blockers used for endotracheal intubation. Analgesia- and sedation-related data from the ED and during the first 48 h of ICU admission included opiates, propofol, benzodiazepines, dexmedetomidine, ketamine, haloperidol, quetiapine, gabapentin, and neuromuscular blockers (i.e., rocuronium, vecuronium, and cisatricurium).

Sedation depth was monitored and recorded according to standard routine care at each site. Deep sedation was defined as: (1) median RASS of − 3 to − 5; or (2) median SAS of 1–3 [15–17, 28] during the first 48 h of care from admission to the ICU. We chose to select a median value to avoid biasing the results with temporarily increased sedation requirements during procedures or imaging that often occur early in patient course.

This period of early sedation was chosen for several reasons. First, early sedation depth appears to be an important contributor to outcome in mechanically ventilated patients. This is demonstrated by several studies which found deep sedation during the initial 48 h of mechanical ventilation to be associated with increased mechanical ventilation duration, mortality, incidence of delirium, and longer lengths of stay [14–16, 19]. Second, this endpoint would allow for an account of the time spent in the ED, which has not been reported before during the COVID-19 pandemic.

Patients were followed until death or hospital discharge. The primary outcome was hospital mortality. Secondary outcomes include ventilator-, ICU-, and hospital-free days.

### Statistical analysis

Descriptive statistics and frequency distributions were used to assess baseline patient characteristics and sedation-related data according to sedation depth. Categorical data were compared with the chi-square test, and continuous data were compared using the independent samples *t*-test or Mann–Whitney *U* test after testing for normality of data. Time (in days) to mortality was assessed with the Kaplan–Meier survival estimate and log-rank test, comparing the early deep sedation and light sedation groups. A second Kaplan–Meier survival estimate was also calculated, which also included patients deeply sedated throughout the first week of ICU care.

To examine the impact of early sedation depth on hospital mortality, a multivariable logistic regression model was used, following recommendations that covariates be selected a priori [29]. The model was adjusted for covariates previously associated with mortality in this cohort: (1) early deep sedation; (2) age; (3) illness severity; (4) indication for mechanical ventilation; and (5) COVID-19 status. All tests were two-tailed and a *p* value of < 0.05 was considered statistically significant. Collinearity was assessed and the model used variables that contributed information that was statistically independent of the other variables in the model.

A post-hoc exploratory analysis was conducted after noting a significantly higher proportion of deeply sedated COVID-19 patients (Table 1). Taking a similar approach to the primary analysis, this secondary analysis analyzed and reported the baseline characteristics and sedation-related data according to COVID-19 status. Additionally, we chose to adjust for race in this post-hoc analysis, given reported outcome differences that had been observed according to race in COVID-19 [30]. To further explore if deep sedation remained independently associated with worse clinical outcomes, a separate multivariable model was conducted on patients positive for COVID-19.

**Table 1:** Characteristics of mechanically ventilated patients based on early sedation depth status

Early sedation depth status			
Baseline characteristics	Light sedation	Deep sedation	*P* value
	(*n* = 108)	(*n* = 283)	
Age (yr)	55.2 (19.4)	56.4 (16.6)	0.53
Gender			
Male, *n* (%)	65 (60.2)	169 (59.7)	0.93
Female, *n* (%)	43 (39.8)	114 (40.3)	
Body mass index (kg/m^2^)	29.5 (8.6)	30.0 (9.6)	0.61
Race, *n* (%)			
White	42 (38.9)	138 (48.8)	0.48
Black	58 (53.7)	120 (42.4)	
Hispanic	3 (2.8)	10 (3.5)	
Asian	1 (0.9)	4 (1.4)	
Native American	0 (0.0)	1 (0.3)	
Other	4 (3.7)	10 (3.5)	
Comorbidities, *n* (%)			
Dementia	8 (7.4)	28 (9.9)	0.45
Diabetes mellitus	30 (27.8)	106 (37.5)	0.07
Cirrhosis	6 (5.6)	13 (4.6)	0.69
CHF	15 (13.9)	51 (18.0)	0.33
ESRD/Dialysis	9 (8.3)	20 (7.1)	0.67
COPD	18 (16.7)	52 (18.4)	0.69
Immunosuppression	4 (3.7)	18 (6.4)	0.31
Malignancy	11 (10.2)	36 (12.7)	0.49
Alcohol abuse	16 (14.8)	27 (9.5)	0.14
Psychiatric*	37 (34.3)	83 (29.3)	0.35
Positive for COVID-19	44 (40.7)	159 (56.2)	0.01
Temperature (Celsius)	36.9 (1.3)	37.0 (1.4)	0.31
Blood pressure (mmHg)			
Systolic	132.7 (34.4)	128.0 (29.8)	0.19
Diastolic	82.0 (24.2)	79.5 (21.6)	0.33
Lactate (mmol/L)	2.0 (1.3–3.1)	2.1 (1.3–3.4)	0.61
Creatinine (mg/dl)	1.1 (0.8–1.8)	1.2 (0.9–2.3)	0.1
Hemoglobin (g/dl)	12.4 (2.5)	12.4 (2.5)	0.85
pH	7.30 (0.12)	7.30 (0.12)	0.95
PaO_2_	137.0 (70.9)	121.0 (76.9)	0.23
PaO_2_:FiO_2_	241.3 (161.0)	184.8 (148.3)	0.04
PaCO_2_	49.4 (16.9)	48.7 (19.9)	0.77
SOFA** (illness severity)	4.5 (2.6)	5.3 (2.5)	0.01
Reason for mechanical ventilation, *n* (%)			
Sepsis	14 (13.0)	44 (15.5)	0.01
Trauma	18 (16.7)	23 (8.1)	
COPD	17 (15.7)	48 (17.0)	
Drug overdose	12 (11.1)	12 (4.2)	
CHF/pulmonary edema	10 (9.3)	22 (7.8)	
Other	13 (12.0)	76 (26.9)	
Cardiac arrest	4 (3.7)	10 (3.5)	
Altered mental status	10 (9.3)	21 (7.4)	
Angioedema	1 (0.9)	5 (1.8)	
Neuromuscular weakness	1 (0.5)	0 (0.0)	
Airway protection	8 (7.4)	22 (7.8)	
Tidal volume (mL/kg PBW)	6.6 (6.1–7.3)	6.5 (6.0–7.3)	0.24
PEEP (cm H_2_O)	6.5 (5.0–10.0)	8.0 (5.0–12.0)	< 0.01
Fraction of inspired oxygen (%)	64.8 (25.9)	74.9 (26.2)	< 0.01
*Process of care variables*			
ED length of stay (h)	5.9 (3.8–8.3)	4.0 (2.5–6.1)	< 0.01
Antibiotics for infection, *n* (%)	50 (47.6)	119 (44.2)	0.56
Vasopressor infusion, *n* (%)	26 (24.3)	72 (25.9)	0.75

CHF, congestive heart failure; ESRD, end-stage renal disease; COPD, chronic obstructive pulmonary disease; SOFA, sequential organ failure assessment score; PEEP, positive end-expiratory pressure; ED, emergency department

Continuous variables are reported as mean (standard deviation) and median (interquartile range)

*Schizophrenia, bipolar disorder, major depression, anxiety

**Modified score, which excludes Glasgow Coma Scale

From prior work regarding the impact of early deep sedation on outcome, we estimated that approximately two-thirds of the cohort would experience early deep sedation, with a mortality of 25% in the early deep sedation group versus 10% in the light sedation group [19]. For 80% power and alpha of 0.05, we estimated a sample size of 219 (82 light sedation, 137 deep sedation) would be required. Based on our prior work regarding mechanically ventilated patients at each site, we were confident that a 6-month enrollment window would be sufficient to accrue the necessary sample size [17, 18, 31–34].

## Results

The data presented here was from the first 6 months of the COVID-19 pandemic and we recognize that practices have evolved dramatically since March of 2020.

### Study population

**Fig. 1:**
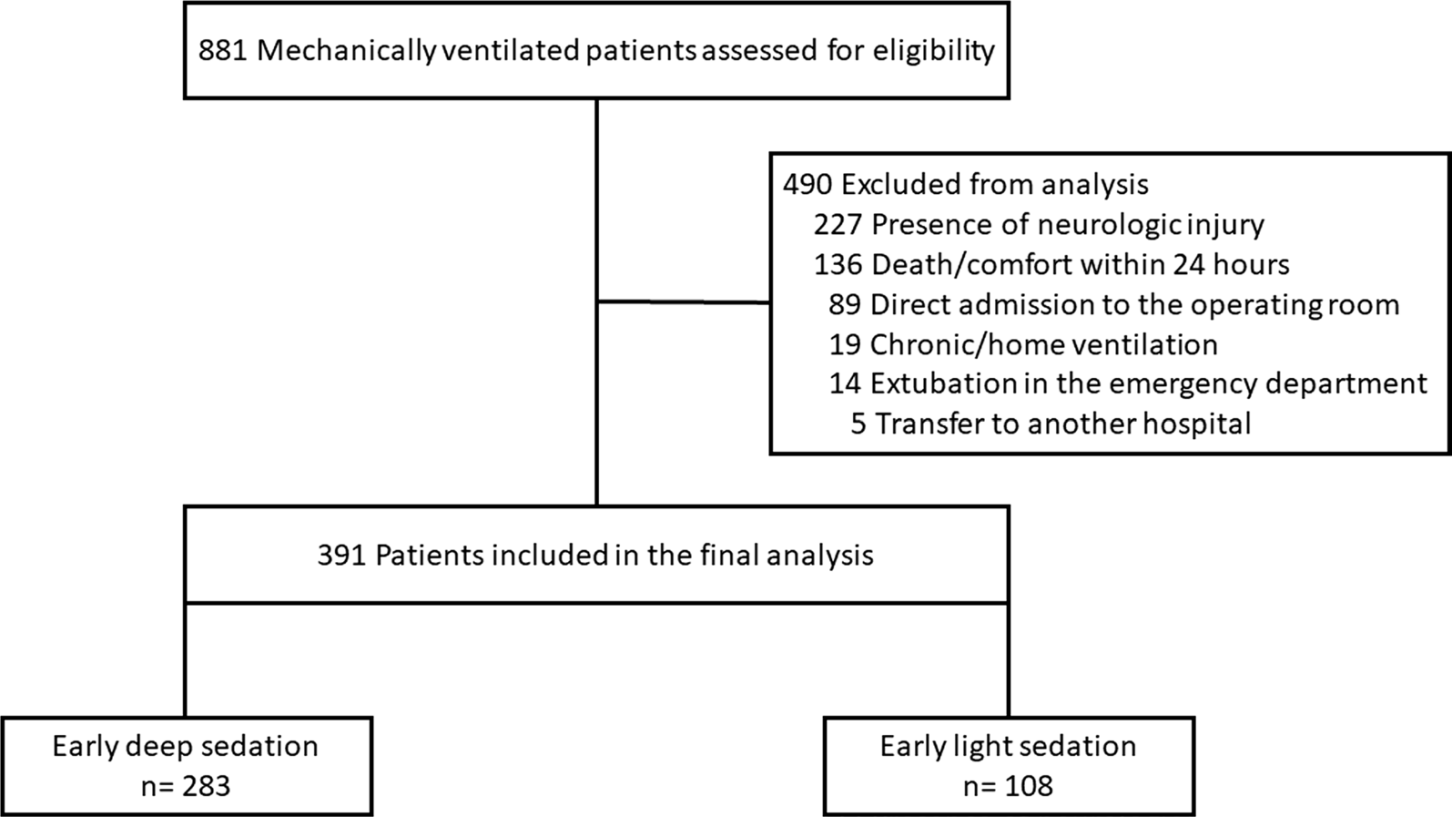
Study flow diagram

Eight hundred eighty-one patients were assessed for eligibility, and 391 comprised the final study population (Fig. 1). Baseline characteristics according to early sedation depth status are in Table 1. Deeply sedated patients had a higher proportion of patients with COVID-19, and a lower partial pressure of arterial oxygenation to fraction of inspired oxygen ratio (PaO2:FiO2).

### Medications administered

Medications used for endotracheal intubation are reported in Additional file 2: Table S1. Sedation variables for the 244 patients that were mechanically ventilated in the ED are in Additional file 3: Table S2. ICU sedation variables for the first 48 h of admission are in Table 2. Deeply sedated patients received higher cumulative doses of fentanyl, propofol, midazolam, hydromorphone, and ketamine when compared to the light sedation group. In addition, deeply sedated patients received neuromuscular blockers more frequently (41.4% vs. 2.1%, *p* < 0.01).

**Table 2:** Sedation variables in the intensive care unit during the first 48 h of admission, according to sedation depth

Early sedation depth status			
	Light sedation (*n* = 108)	Deep sedation (*n* = 283)	*p*
*Sedative drug*			
Propofol			
* n* (%)	85 (78.7)	222 (78.4)	0.96
Cumulative dose (mg)	1526 (600–4914)	4047 (1507–8109)	< 0.01
Midazolam			
* n* (%)	35 (32.4)	115 (40.6)	0.14
Cumulative dose (mg)	12.0 (3.0–54.0)	19.0 (5.0–152.0)	0.09
Dexmedetomidine			
* n* (%)	50 (46.3)	93 (32.9)	0.01
Cumulative dose (mcg/kg)	4.6 (2.0–9.5)	7.0 (2.1–17.8)	0.08
Lorazepam			
* n* (%)	11 (10.2)	27 (9.5)	0.85
Cumulative dose (mg)	3.0 (1.0–12.0)	2.0 (1.0–3.0)	0.32
Ketamine			
* n* (%)	10 (9.3)	38 (13.4)	0.26
Cumulative dose (mg)	87.5 (50.0–250.0)	675.0 (187.5–2050.0)	< 0.01
Haloperidol			
* n* (%)	9 (8.3)	13 (4.6)	0.15
Cumulative dose (mg)	5.0 (5.0–10.0)	5.0 (5.0–10.0)	0.95
Quetiapine			
* n* (%)	4 (3.7)	12 (4.2)	0.81
Cumulative dose (mg)	37.5 (25.0–237.5)	200.0 (31.3–287.5)	0.91
Gabapentin			
* n* (%)	11 (10.2)	11 (3.9)	0.02
Cumulative dose (mg)	600.0 (300.0–2100.0)	1200 (300–2100)	0.33
*Analgesic drug*			
Fentanyl			
* n* (%)	92 (85.2)	240 (84.8)	0.93
Cumulative dose (mcg)	3175 (1206–6330)	3950 (1600–6950)	0.09
Hydromorphone			
* n* (%)	12 (11.1)	49 (17.3)	0.13
Cumulative dose (mg)	2.5 (1.0–17.8)	9.0 (3.0–69.0)	0.04
Oxycodone			
* n* (%)	18 (16.7)	28 (9.9)	0.06
Cumulative dose (mg)	17.5 (10.0–32.5)	20.0 (10.0–40.0)	0.96
Morphine			
* n* (%)	1 (0.9)	7 (2.5)	0.33
Cumulative dose (mg)	2.0 (NA)	6.5 (2.0–12.8)	0.57
Neuromuscular blocker, *n* (%)	4 (2.1)	84 (41.4)	< 0.01
RASS level ICU day 1	− 1 (− 2 to − 0)	− 3 (− 4 to − 2)	< 0.01
SAS level ICU day 1	4 (4–4)	3 (2–4)	< 0.01
RASS level ICU day 2	− 1 (− 2 to 0)	− 3 (− 5 to − 2)	< 0.01
SAS level ICU day 2	4 (4–4)	3 (3–4)	< 0.01
RASS level ICU days 3–7	− 1 (− 2 to 0)	− 3 (− 4 to − 1)	< 0.01
SAS level ICU days 3–7	4 (4–4)	3 (3–4)	< 0.01
Deep sedation ICU days 3–7, *n* (%)*	14 (18.4)	128 (53.8)	< 0.01
Deep sedation until death, *n* (%)	0 (0.0)	94 (33.2)	< 0.01

ICU, intensive care unit; RASS, Richmond Agitation-Sedation Scale

^*^Denominator is 314 (238 deep sedation group and 76 light sedation group)

### Depth of sedation

Deep sedation occurred in 72.4% of all patients (both COVID-19 and non-COVID-19 cohorts) during the first 48 h. Sedation levels differed significantly (*p* < 0.01 for each) between the deep sedation and light sedation groups during this period. This difference persisted through the first seven days of mechanical ventilation (Table 2), such that 128 (53.8%) patients in the deep sedation group experienced deep sedation during the first week of ICU care, as compared to 14 (18.4%) patients in the light sedation group, *p* < 0.01. Ninety-four (33.2%) deeply sedated patients remained deeply sedated until death, compared to 0 (0.0%) patients in the light sedation group, *p* < 0.01.

### Subgroup analyses

Baseline characteristics according to COVID-19 status are in Additional file 4: Table S3. ED sedation variables are in Additional file 5: Table S4, and ICU sedation variables from the first 48 h are in Table 3. No significant differences in medication doses were observed in the ED. In the ICU, COVID-19 patients received significantly higher cumulative doses of fentanyl, propofol, midazolam, hydromorphone, and ketamine when compared to non-COVID patients. COVID-19 patients also received neuromuscular blockers more frequently than non-COVID patients in the ICU (41.4% vs. 2.1%, *p* < 0.01). COVID-19 patients experienced deep sedation more frequently early and throughout the first week of ICU care (*p* < 0.01 for all). Seventy-eight (38.4%) COVID patients remained deeply sedated until death, compared to 16 (8.5%) non-COVID patients.

**Table 3:** Sedation variables in the intensive care unit during the first 48 h of admission, according to COVID status

COVID status			
Drug	Non-COVID (*n* = 188)	COVID (*n* = 203)	*p*
Fentanyl			
* n* (%)	148 (78.7)	184 (90.6)	< 0.01
Cumulative dose (mcg)	1562 (509–4063)	5350 (3275–8050)	< 0.01
Propofol			
* n* (%)	143 (76.1)	164 (80.8)	0.26
Cumulative dose (mg)	2324 (1021–6443)	4047 (1227–8127)	0.02
Midazolam			
* n* (%)	36 (19.1)	114 (56.2)	< 0.01
Cumulative dose (mg)	4.0 (2.0–30.0)	31.5 (5.0–155.0)	< 0.01
Dexmedetomidine			
* n* (%)	91 (48.4)	52 (25.6)	< 0.01
Cumulative dose (mcg/kg)	5.3 (2.2–15.4)	5.3 (1.6–13.1)	0.67
Lorazepam			
* n* (%)	17 (9.0)	21 (10.3)	0.66
Cumulative dose (mg)	2.0 (1.5–11.0)	2.0 (1.0–3.5)	0.37
Hydromorphone			
* n* (%)	38 (20.2)	23 (11.3)	0.02
Cumulative dose (mg)	4.5 (2.0–11.0)	71.0 (9.0–108.0)	< 0.01
Oxycodone			
* n* (%)	28 (14.9)	18 (8.9)	0.07
Cumulative dose (mg)	17.5 (10.0–37.5)	20.0 (10.0–35.0)	0.76
Morphine			
* n* (%)	1 (0.5)	7 (3.4)	0.04
Cumulative dose (mg)	8.0 (NA)	3.5 (2.0–12.8)	0.86
Ketamine			
* n* (%)	16 (8.5)	32 (15.8)	0.03
Cumulative dose (mg)	92.5 (50.0–350.0)	950.0 (234.0–2050.0)	< 0.01
Haloperidol			
* n* (%)	14 (7.4)	8 (3.9)	0.13
Cumulative dose (mg)	5.0 (5.0–11.3)	5.0 (5.0–8.8)	0.37
Quetiapine			
* n* (%)	5 (2.7)	11 (5.4)	0.17
Cumulative dose (mg)	50.0 (37.5–300.0)	200.0 (25.0–250.0)	0.91
Gabapentin			
* n* (%)	13 (6.9)	9 (4.4)	0.29
Cumulative dose (mg)	600.0 (300.0–2100.0)	800.0 (350.0–2100.0)	0.85
Neuromuscular blocker			
* n* (%)	4 (2.1)	84 (41.4)	< 0.01
RASS level ICU day 1	− 2 (− 3 to − 1)	− 3 (− 4 to − 2)	< 0.01
SAS level ICU day 1	3 (3–4)	2 (1–4)	< 0.01
RASS level ICU day 2	− 1 (− 2 to 0)	− 3 (− 5 to − 2)	< 0.01
SAS level ICU day 2	4 (3–4)	3 (1–3)	< 0.01
RASS level ICU days 3–7	0 (− 2 to 0)	− 3 (− 5 to − 2)	< 0.01
SAS level ICU days 3–7	4 (3–4)	2 (1–3)	< 0.01
Deep sedation ICU day 1, *n* (%)	73 (38.8)	118 (58.1)	< 0.01
Deep sedation ICU day 2, *n* (%)*	43 (25.9)	117 (60.6)	< 0.01
Deep sedation ICU days 3–7, *n* (%)**	25 (19.4)	109 (61.6)	< 0.01
Deep sedation until death, *n* (%)	16 (8.5)	78 (38.4)	< 0.01

ICU, intensive care unit; RASS, Richmond Agitation-Sedation Scale

*Denominator is 359 (193 COVID group and 166 non-COVID group)

**Denominator is 314 (185 COVID group and 129 non-COVID group)

### Clinical outcomes

**Fig. 2:**
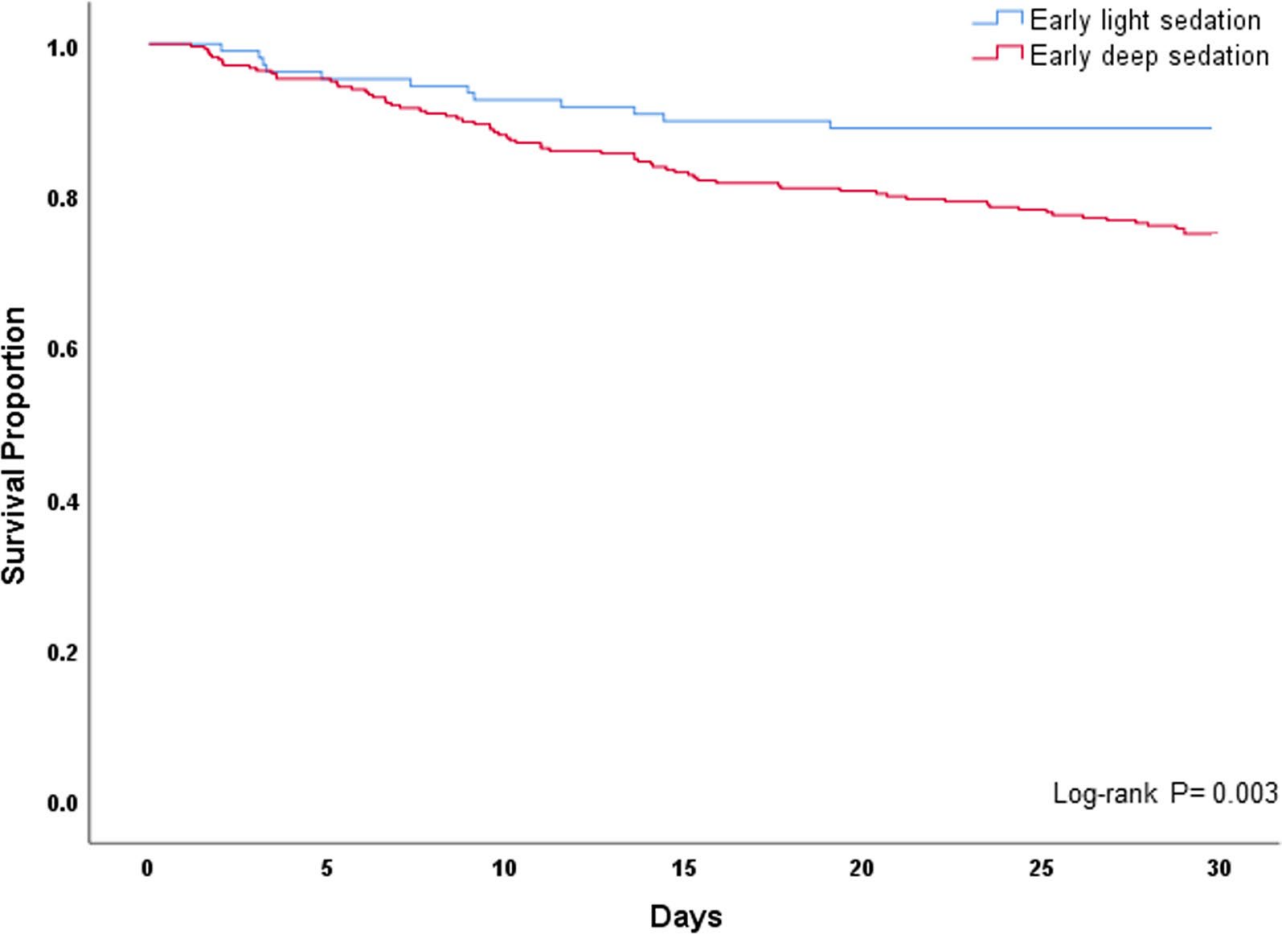
Kaplan–Meier survival curves of 391 mechanically ventilated patients comparing early deep (*n* = 283) and light sedation groups (*n* = 108)

Table 4 shows that in the unadjusted analysis of clinical outcomes according to sedation depth, deep sedation patients experienced fewer ventilator-, ICU-, and hospital-free days, and greater mortality (30.4% versus 11.1%) when compared to light sedation (*p* < 0.01 for all). On Kaplan–Meier analysis, survival diverged significantly between the early deep sedation and light sedation groups (log-rank *p* < 0.01, Fig. 2). After adjusting for confounders (Additional file 6: Table S5), early deep sedation remained significantly associated with higher mortality (adjusted OR 3.44; 95% CI 1.65–7.17; *p* < 0.01).

**Table 4:** Unadjusted analysis of clinical outcomes according to early sedation depth

Outcome	Light sedation (*n* = 108)	Deep sedation (*n* = 283)	OR or between-group difference (95% CI)	*p*
Ventilator-free days	20.7 (9.6)	14.7 (11.4)	6.04 (3.60–8.48)	< 0.01
ICU-free days	18.3 (9.9)	12.1 (11.0)	6.20 (3.82–8.57)	< 0.01
Hospital-free days	13.8 (10.3)	8.0 (9.6)	5.74 (3.56–7.92)	< 0.01
Mortality, *n* (%)	12 (11.1)	86 (30.4)	3.49 (1.82–6.70)	< 0.01

ICU, intensive care unit; OR, odds ratio; CI, confidence interval

In the subgroup analysis (Additional file 7: Table S6), similar unadjusted clinical outcomes according to COVID status were seen, such that COVID patients experienced fewer ventilator-, ICU-, and hospital-free days (*p* < 0.01 for all). Mortality was 41.4% in COVID patients versus 7.4% in non-COVID patients (*p* < 0.01). After adjusting for confounders (Additional file 7: Table S6), early deep sedation remained significantly associated with higher mortality (adjusted OR 2.76; 95% CI 1.26–6.06; *p* < 0.01), though illness severity remained an important variable in this analysis.

## Discussion

Given the importance of high-quality supportive therapies in critical illness, the potential impact of early sedation depth on clinical outcomes, and a dearth of early sedation data in the COVID-19 era, we conducted the COVID-SED study to characterize ED and early ICU sedation practices during the COVID-19 pandemic and assess the impact of early deep sedation on clinical outcomes. We found that over 70% of mechanically ventilated patients experienced early deep sedation, with significant differences in cumulative medication doses and neuromuscular blockade. In addition, early deep sedation frequently persisted throughout the first week of mechanical ventilation and was negatively associated with outcome.

Our most important finding was an association between early deep sedation and worse clinical outcomes. Early deep sedation was associated with fewer ventilator-, ICU-, and hospital-free days, and increased hospital mortality. These results remained significant after adjustment for confounders and were consistent in the subgroup of patients with COVID. Our findings are supported by prior work in the pre-COVID era, which showed the negative relationship between early deep sedation and patient-centered clinical outcomes [14–19]. Additionally, these findings are congruent with a recent analysis that examined the impact of deep sedation in a comparison of patients with COVID-associated ARDS with non-COVID historical controls [20]. The findings of the COVID-SED Study are further support of a guideline- and protocol-driven approach to sedation management, regardless of COVID status [35].

A second important finding is the characterization of sedation practices during the first wave of the COVID pandemic. Sedation in the ED was similar to prior work, suggesting that the COVID era influenced ED-based sedation little [17]. However, compared with pre-COVID work, sedation in the ICU saw an increased use and higher doses of fentanyl, benzodiazepines, and ketamine, which appeared largely driven by COVID status [17]. The occurrence rate of 72.4% of early deep sedation is also higher than that seen in recent pre-COVID publications and further highlights the rapidly adopted changes in sedation practice that occurred with the COVID pandemic [17, 19]. These findings are consistent with prior reports that documented high sedative and neuromuscular blockade use in COVID patients[20, 21, 36–39]. Further, our findings highlight the static nature in the approach to sedation in the early deep sedation group: (1) > 50% experienced deep sedation throughout the first week of mechanical ventilation; and (2) 33% were deeply sedated until death. While not formally measured in this study, these results further suggest low adherence to the ABCDEF bundle, congruent with a prior international point prevalence study on ICU patients with COVID [40].

Another important finding involves the sedation observed in non-COVID patients. Given the significant changes in supportive care observed during the onset of the COVID pandemic, it is reasonable to hypothesize that the care of non-COVID patients would have been altered as well. However, when compared to prior work, patients in the non-COVID group experienced sedation management, early deep sedation, and clinical outcomes similar to that seen in the pre-COVID era [17]. This suggests that the observed changes in the standards of critical care were isolated to COVID patients and further highlight the importance of continued assessments into protocol-driven supportive care in this cohort.

This work has several important limitations. This is one of the first studies examining the impact of sedation depth on clinical outcomes during the COVID pandemic, yet it is relatively small and therefore prone to bias. As a two-center study, it is possible that these data are not truly representative and lack external validity. All data were obtained retrospectively and therefore subject to potential inaccuracies in routine documentation. The study design can only inform on association and not causation, and the ability to control for confounding is limited. Furthermore, we did not collect data on standardized pain monitoring scores due to variability in practice between different units and different hospitals in our study group. This is a potential significant confounder as inadequate analgesia may have played a role in greater sedative requirements.

In performing this study, we collected data regarding indication for mechanical ventilation. We acknowledge that adjudicating this variable is not straightforward and can be subjective. For example, a patient with COPD can be infected and therefore have sepsis and either could be the cause for respiratory failure. Recognizing that both acute respiratory failure and ARDS are broad syndromes that can have myriad causes, we recognize that the categorical definition of this variable is a limitation of this study.

Deep sedation, and therefore the possible the need for it, overlapped with COVID status, and may also have been a marker of illness severity and the presence of ARDS. Moreover, deep sedation may be necessary to overcome the potentially injurious respiratory drive seen in COVID ARDS[41]. Increased deep sedation incidence and duration may also be the result of increased sedation protocol violations during the COVID era. Our results are consistent with prior literature regarding the impact of early deep sedation on outcomes, and the association between deep sedation and mortality remained strong after adjusting for SOFA (which includes oxygenation). While this is encouraging and lends face validity, the relationship between early deep sedation and disease severity is difficult to truly separate through statistical methods. As such, these results should be viewed as hypothesis-generating. These data were collected during the first 6 months of the COVID pandemic, and therefore may not reflect rapidly evolving COVID era sedation practices. However, this work highlights the importance of adhering to proven ICU principles, such as ventilator management to avoid asynchrony and judicious sedation use, and are informative for the potential of persistent COVID-19 or future viral pandemics [42]. Finally, depressed mental status and deeper sedation levels may have been secondary to COVID or concomitant intracranial abnormalities, as opposed to sedation management [43]. We did not collect any neuroimaging data for this study, leaving this an unaddressed confounder.

## Conclusion

The management of sedation for mechanically ventilated patients in the ICU has been impacted by the COVID pandemic. Early deep sedation is common, especially among COVID-19 patients, and independently associated with worse clinical outcomes. A protocol-driven approach to sedation, targeting light sedation as early as possible, should continue to remain the default approach.

## Supplementary Information

**Additional file 1**. STROBE checklist.

**Additional file 2: Table S1**. Medications used for endotracheal intubation based on early sedation depth status.

**Additional file 3: Table S2**. Sedation variables, according to sedation depth, for the 244 patients that received mechanical ventilation in the emergency department.

**Additional file 4: Table S3**. Characteristics of mechanically ventilated patients based on presence of coronavirus disease.

**Additional file 5: Table S4**. Sedation variables for the 244 patients that received mechanical ventilation in the emergency department, according to COVID status.

**Additional file 6: Table S5**. Results of the multivariable logistic regression analysis for the primary outcome of mortality.

**Additional file 7: Table S6**. (A) Unadjusted clinical outcomes according to COVID status, and (B) results of the multivariable logistic regression analysis for mortality in the subgroup of patients (*n* = 203) that were positive for COVID.

## Data Availability

The datasets used and/or analyzed during the current study are available from the corresponding author on reasonable request.

## Abbreviations

ARDS: Acute respiratory distress syndrome
COVID: Coronavirus disease
ED: Emergency department
FiO2: Fraction of inspired oxygen
HRPO: Human Research Protection Office
ICU: Intensive care unit
IRB: Institutional Review Board
OR: Operating room
PaO2: Partial pressure of arterial oxygenation
RASS: Richmond Agitation-Sedation Scale
SAS: Sedation-Agitation Scale
SOFA: Sequential organ failure assessment
STROBE: Strengthening Reporting of Observational Studies in Epidemiology

## Declarations

### Guarantor statement

BMF takes responsibility for the content of the manuscript as a whole.

### Notification of prior abstract publication/presentation

Partial results from this study were presented at the Society of Critical Care Medicine (April, 2022) annual congress.

## References

[1] Harhay MO, Wagner J, Ratcliffe SJ, Bronheim RS, Gopal A, Green S, Cooney E, Mikkelsen ME, Kerlin MP, Small DS (2014). Outcomes and statistical power in adult critical care randomized trials. Am J Respir Crit Care Med.

[2] Morris PE, Goad A, Thompson C, Taylor K, Harry B, Passmore L, Ross A, Anderson L, Baker S, Sanchez M (2008). Early intensive care unit mobility therapy in the treatment of acute respiratory failure. Crit Care Med.

[3] Pronovost P, Needham D, Berenholtz S, Sinopoli D, Chu H, Cosgrove S, Sexton B, Hyzy R, Welsh R, Roth G (2006). An intervention to decrease catheter-related bloodstream infections in the ICU. N Engl J Med.

[4] Network ARDS, Wheeler A, Bernard G, Thompson B, Hayden D, DeBoisblanc B (2006). Comparison of two fluid-management strategies in acute lung injury. N Engl J Med.

[5] Acute Respiratory Distress Syndrome Network (2000). Ventilation with lower tidal volumes as compared with traditional tidal volumes for acute lung injury and the acute respiratory distress syndrome. N Engl J Med.

[6] Girard TD, Kress JP, Fuchs BD, Thomason JW, Schweickert WD, Pun BT, Taichman DB, Dunn JG, Pohlman AS, Kinniry PA (2008). Efficacy and safety of a paired sedation and ventilator weaning protocol for mechanically ventilated patients in intensive care (Awakening and Breathing Controlled trial): a randomised controlled trial. Lancet.

[7] Kollef MH, Levy NT, Ahrens TS, Schaiff R, Prentice D, Sherman G (1998). The use of continuous iv sedation is associated with prolongation of mechanical ventilation. Chest.

[8] Kress JP, O'Connor MF, Pohlman AS, Olson D, Lavoie A, Toledano A, Hall JB (1996). Sedation of critically ill patients during mechanical ventilation. A comparison of propofol and midazolam. Am J Respir Crit Care Med.

[9] Kress JP, Pohlman AS, O'Connor MF, Hall JB (2000). Daily interruption of sedative infusions in critically ill patients undergoing mechanical ventilation. N Engl J Med.

[10] Mehta S, Burry L, Cook D, Fergusson D, Steinberg M, Granton J, Herridge M, Ferguson N, Devlin J, Tanios M (2012). Daily sedation interruption in mechanically ventilated critically ill patients cared for with a sedation protocol: a randomized controlled trial. JAMA.

[11] Quenot J-P, Ladoire S, Devoucoux F, Doise J-M, Cailliod R, Cunin N, Aubé H, Blettery B, Charles PE (2007). Effect of a nurse-implemented sedation protocol on the incidence of ventilator-associated pneumonia. Crit Care Med.

[12] Reade MC, Finfer S (2014). Sedation and delirium in the intensive care unit. N Engl J Med.

[13] Shehabi Y (2018). The golden hours of ICU sedation: the clock is ticking. Crit Care Med.

[14] Shehabi Y, Bellomo R, Kadiman S, Ti LK, Howe B, Reade MC, Khoo TM, Alias A, Wong Y-L, Mukhopadhyay A (2018). Sedation intensity in the first 48 hours of mechanical ventilation and 180-day mortality: a multinational prospective longitudinal cohort study. Crit Care Med.

[15] Shehabi Y, Bellomo R, Reade MC, Bailey M, Bass F, Howe B, McArthur C, Seppelt IM, Webb S, Weisbrodt L (2012). Early intensive care sedation predicts long-term mortality in ventilated critically ill patients. Am J Respir Crit Care Med.

[16] Shehabi Y, Chan L, Kadiman S, Alias A, Ismail WN, Tan MATI, Khoo TM, Ali SB, Saman MA, Shaltut A (2013). Sedation depth and long-term mortality in mechanically ventilated critically ill adults: a prospective longitudinal multicentre cohort study. Intensive Care Med.

[17] Fuller BM, Roberts BW, Mohr NM, Knight WA, Adeoye O, Pappal RD, Marshall S, Alunday R, Dettmer M, Goyal M (2019). The ED-SED study: a multicenter, prospective cohort study of practice patterns and clinical outcomes associated with emergency department sedation for mechanically ventilated patients. Crit Care Med.

[18] Stephens RJ, Ablordeppey E, Drewry AM, Palmer C, Wessman BT, Mohr NM, Roberts BW, Liang SY, Kollef MH, Fuller BM (2017). Analgosedation practices and the impact of sedation depth on clinical outcomes among patients requiring mechanical ventilation in the ED: a cohort study. Chest.

[19] Stephens RJ, Dettmer MR, Roberts BW, Ablordeppey E, Fowler SA, Kollef MH, Fuller BM (2017). Practice patterns and outcomes associated with early sedation depth in mechanically ventilated patients: a systematic review and meta-analysis. Crit Care Med.

[20] Wongtangman K, Santer P, Wachtendorf LJ, Azimaraghi O, Kassis EB, Teja B, Murugappan KR, Siddiqui S, Eikermann M (2021). Association of sedation, coma, and in-hospital mortality in mechanically ventilated patients with coronavirus disease 2019–related acute respiratory distress syndrome: a retrospective cohort study. Crit Care Med.

[21] Pun BT, Badenes R, La Calle GH, Orun OM, Chen W, Raman R, Simpson B-GK, Wilson-Linville S, Olmedillo BH, de la Cueva AV (2021). Prevalence and risk factors for delirium in critically ill patients with COVID-19 (COVID-D): a multicentre cohort study. Lancet Respir Med.

[22] Zampieri FG, Bastos LSL, Soares M, Salluh JI, Bozza FA (2021). The association of the COVID-19 pandemic and short-term outcomes of non-COVID-19 critically ill patients: an observational cohort study in Brazilian ICUs. Intensive Care Med.

[23] von Elm E, Altman DG, Egger M, Pocock SJ, Gotzsche PC, Vandenbroucke JP (2007). The strengthening the reporting of observational studies in epidemiology (STROBE) statement: guidelines for reporting observational studies. Ann Internal Med.

[24] Harris PA, Taylor R, Minor BL, Elliott V, Fernandez M, O'Neal L, McLeod L, Delacqua G, Delacqua F, Kirby J (2019). The REDCap consortium: building an international community of software platform partners. J Biomed Inform.

[25] Harris PA, Taylor R, Thielke R, Payne J, Gonzalez N, Conde JG (2009). Research electronic data capture (REDCap)—a metadata-driven methodology and workflow process for providing translational research informatics support. J Biomed Inform.

[26] Vincent J, Moreno R, Takala J, Willatts S, De Mendonça A, Bruining H, Reinhart C, Suter P, Thijs L (1996). The SOFA (Sepsis-related Organ Failure Assessment) score to describe organ dysfunction/failure. Intensive Care Med.

[27] Vincent J-L, De Mendonça A, Cantraine F, Moreno R, Takala J, Suter PM, Sprung CL, Colardyn F, Blecher S (1998). Use of the SOFA score to assess the incidence of organ dysfunction/failure in intensive care units: results of a multicenter, prospective study. Crit Care Med.

[28] Tanaka LMS, Azevedo LCP, Park M, Schettino G, Nassar AP, Réa-Neto A, Tannous L, de Souza-Dantas VC, Torelly A, Lisboa T (2014). Early sedation and clinical outcomes of mechanically ventilated patients: a prospective multicenter cohort study. Crit Care.

[29] Maslove DM, Leisman DE (2019). Causal inference from observational data: New guidance from pulmonary, critical care, and sleep journals. Crit Care Med.

[30] Qureshi AI, Baskett WI, Huang W, Shyu D, Myers D, Lobanova I, Naqvi SH, Thompson VS, Shyu CR (2021). Effect of race and ethnicity on in-hospital mortality in patients with COVID-19. Ethn Dis.

[31] Fuller BM, Ferguson IT, Mohr NM, Drewry AM, Palmer C, Wessman BT, Ablordeppey E, Keeperman J, Stephens RJ, Briscoe CC (2017). Lung-protective ventilation initiated in the emergency department (LOV-ED): a quasi-experimental, before-after trial. Ann Emerg Med.

[32] Fuller BM, Ferguson IT, Mohr NM, Drewry AM, Palmer C, Wessman BT, Ablordeppey E, Keeperman J, Stephens RJ, Briscoe CC (2017). A quasi-experimental, before-after trial examining the impact of an emergency department mechanical ventilator protocol on clinical outcomes and lung-protective ventilation in acute respiratory distress syndrome. Crit Care Med.

[33] Fuller BM, Mohr NM, Miller CN, Deitchman AR, Levine BJ, Castagno N, Hassebroek EC, Dhedhi A, Scott-Wittenborn N, Grace E (2015). Mechanical ventilation and ARDS in the ED: a multicenter, observational, prospective, cross-sectional study. Chest.

[34] Pappal RD, Roberts BW, Mohr NM, Ablordeppey E, Wessman BT, Drewry AM, Winkler W, Yan Y, Kollef MH, Avidan MS (2021). The ED-AWARENESS study: a prospective, observational cohort study of awareness with paralysis in mechanically ventilated patients admitted from the emergency department. Ann Emerg Med.

[35] Devlin JW, Pandharipande PP (2021). Do our sedation practices contribute to increased mortality in coronavirus disease 2019–related acute respiratory distress syndrome?. Crit Care Med.

[36] Tapaskar N, Colon Hidalgo D, Koo G, Shingada K, Rao S, Rodriguez R, Alcantar D, Espinoza Barrera D, Lee R, Rameshkumar N (2021). Sedation usage in COVID-19 acute respiratory distress syndrome: a multicenter study. Ann Pharmacother.

[37] Flinspach AN, Booke H, Zacharowski K, Balaban Ü, Herrmann E, Adam EH (2021). High sedation needs of critically ill COVID-19 ARDS patients—A monocentric observational study. PLoS ONE.

[38] Khan SH, Lindroth H, Perkins AJ, Jamil Y, Wang S, Roberts S, Farber M, Rahman O, Gao S, Marcantonio ER (2020). Delirium incidence, duration, and severity in critically ill patients with coronavirus disease 2019. Crit Care Explor.

[39] Balakrishna A, Walsh EC, Hamidi A, Berg S, Austin D, Pino RM, Hanidziar D, Chang MG, Bittner EA (2021). An examination of sedation requirements and practices for mechanically ventilated critically ill patients with COVID-19. Am J Health Syst Pharm AJHP.

[40] Liu K, Nakamura K, Katsukawa H, Elhadi M, Nydahl P, Ely EW, Kudchadkar SR, Takahashi K, Inoue S, Lefor AK (2021). ABCDEF Bundle and supportive ICU practices for patients with coronavirus disease 2019 infection: An international point prevalence study. Crit Care Explor.

[41] Esnault P, Cardinale M, Hraiech S, Goutorbe P, Baumstrack K, Prud'homme E, Bordes J, Forel JM, Meaudre E, Papazian L (2020). High respiratory drive and excessive respiratory efforts predict relapse of respiratory failure in critically ill patients with COVID-19. Am J Respir Crit Care Med.

[42] Chanques G, Constantin JM, Devlin JW, Ely EW, Fraser GL, Gelinas C, Girard TD, Guerin C, Jabaudon M, Jaber S (2020). Analgesia and sedation in patients with ARDS. Intensive Care Med.

[43] Kremer S, Lersy F, de Sèze J, Ferré J-C, Maamar A, Carsin-Nicol B, Collange O, Bonneville F, Adam G, Martin-Blondel G (2020). Brain MRI findings in severe COVID-19: a retrospective observational study. Radiology.

