# Supplementary material for: A dual-center cohort study on the association between early deep sedation and clinical outcomes in mechanically ventilated patients during the COVID-19 pandemic: The COVID-SED study
Source: Crit Care. 2022 Jun 15;26:179. doi: 10.1186/s13054-022-04042-9 (PMC9198202; doi:10.1186/s13054-022-04042-9)
Supplement: Supplementary file 2 — Additional file 2: Table S1. Medications used for endotracheal intubation based on early sedation depth status. [file 13054_2022_4042_MOESM2_ESM.docx]

**Additional file 2: Table S1.** Medications used for endotracheal intubation based on early sedation depth status.

| **Early Sedation Depth Status** | | | |
| --- | --- | --- | --- |
| **Medication** | **Light Sedation**  **(n= 108)** | **Deep Sedation**  **(n= 283)** | ***P* value** |
| Location of intubation  ED, n (%)  Prehospital, n (%)  Transferring facility, n (%)  Intensive care unit, n (%) | 60 (67.6)  8 (7.4)  22 (20.4)  18 (16.7) | 114 (55.6)  25 (8.8)  57 (20.1)  87 (30.7) | 0.02 |
| Neuromuscular blocker, n (%)*^a^*  Succinylcholine  Rocuronium  Vecuronium  None | 40 (40.0)  45 (45.0)  2 (2.0)  13 (13.0) | 82 (31.1)  147 (55.7)  4 (1.5)  31 (11.7) | 0.32 |
| Induction agent, n (%)*^b^*  Etomidate  Ketamine  Propofol  Midazolam  None | 52 (51.5)  35 (34.7)  4 (4.0)  5 (5.0)  5 (5.0) | 121 (45.8)  83 (31.4)  24 (9.1)  8 (3.0)  28 (10.6) | 0.16 |

*^a^*Excludes 27 patients with missing data

*^b^*Excludes 26 patients with missing data
