# Supplementary material for: A dual-center cohort study on the association between early deep sedation and clinical outcomes in mechanically ventilated patients during the COVID-19 pandemic: The COVID-SED study
Source: Crit Care. 2022 Jun 15;26:179. doi: 10.1186/s13054-022-04042-9 (PMC9198202; doi:10.1186/s13054-022-04042-9)
Supplement: Supplementary file 5 — Additional file 5: Table S4. Sedation variables for the 244 patients that received mechanical ventilation in the emergency department, according to COVID status. [file 13054_2022_4042_MOESM5_ESM.docx]

**Additional file 5: Table S4.** Sedation variables for the 244 patients that received mechanical ventilation in the emergency department, according to COVID status.

| **COVID Status** | | | |
| --- | --- | --- | --- |
| **Drug** | **Non-COVID**  **(n= 188)** | **COVID**  **(n= 56)** | ***p*** |
| Fentanyl  n (%)  Cumulative dose (mcg) | 136 (72.3)  300 (102.5 – 450.0) | 47 (83.9)  200 (150 – 350.0) | 0.08  0.36 |
| Propofol  n (%)  Cumulative dose (mg) | 127 (67.6)  433.5 (188.3 – 813.1) | 33 (58.9)  267.3 (149.9 – 559.4) | 0.23  0.11 |
| Midazolam  n (%)  Cumulative dose (mg) | 45 (23.9)  5.0 (3.0 – 9.5) | 12 (21.4)  6.5 (3.5 – 11.5) | 0.70  0.24 |
| Dexmedetomidine  n (%)  Cumulative dose (mcg) | 15 (8.0)  1.6 (1.0 – 2.2) | 3 (5.4)  2.7 (0.4 – NA) | 0.51  0.36 |
| Ketamine  n (%)  Cumulative dose (mg) | 15 (8.0)  100 (50.0 – 200) | 14 (25.0)  155 (90.0 – 200) | <0.01  0.56 |
| Lorazepam  n (%)  Cumulative dose (mg) | 25 (13.3)  2.0 (1.0 – 4.0) | 2 (3.6)  1.5 (1.0 – NA) | 0.04  0.37 |
| Hydromorphone  n (%)  Cumulative dose (mg) | 6 (3.2)  1.0 (1.0 – 2.0) | 1 (1.8)  1.0 (NA) | 0.58  0.86 |
| Haloperidol  n (%)  Cumulative dose (mg) | 6 (3.2)  6.5 (5.0 – 10.5) | 0 (0.0)  NA | 0.18  NA |
| Neuromuscular blocker, n (%) | 3 (1.6) | 5 (8.9) | <0.01 |
