# Supplementary material for: A dual-center cohort study on the association between early deep sedation and clinical outcomes in mechanically ventilated patients during the COVID-19 pandemic: The COVID-SED study
Source: Crit Care. 2022 Jun 15;26:179. doi: 10.1186/s13054-022-04042-9 (PMC9198202; doi:10.1186/s13054-022-04042-9)
Supplement: Supplementary file 6 — Additional file 6: Table S5. Results of the multivariable logistic regression analysis for the primary outcome of mortality. [file 13054_2022_4042_MOESM6_ESM.docx]

**Additional file 6: Table S5.** Results of the multivariable logistic regression analysis for the primary outcome of mortality.

| **Variable** | **aOR** | **95% CI** | ***P* value** |
| --- | --- | --- | --- |
| Early deep sedation | 3.44 | 1.65 – 7.17 | <0.01 |
| Age | 1.03 | 1.01 – 1.05 | <0.01 |
| Positive for COVID-19 | 6.43 | 3.39 – 12.19 | <0.01 |
| Total SOFA Score | 1.20 | 1.08 – 1.34 | 0.01 |
| Indication for mechanical ventilation | 1.36 | 0.49 – 3.76 | 0.55 |

SOFA: sequential organ failure assessment; aOR: adjusted odds ration; CI: confidence interval
