# Supplementary material for: A dual-center cohort study on the association between early deep sedation and clinical outcomes in mechanically ventilated patients during the COVID-19 pandemic: The COVID-SED study
Source: Crit Care. 2022 Jun 15;26:179. doi: 10.1186/s13054-022-04042-9 (PMC9198202; doi:10.1186/s13054-022-04042-9)
Supplement: Supplementary file 7 — Additional file 7: Table S6. (A) Unadjusted clinical outcomes according to COVID status, and (B) results of the multivariable logistic regression analysis for mortality in the subgroup of patients (n = 203) that were positive for COVID. [file 13054_2022_4042_MOESM7_ESM.doc]

**Additional file 7: Table S6.** (A) Unadjusted clinical outcomes according to COVID status, and (B) results of the multivariable logistic regression analysis for mortality in the subgroup of patients (n= 203) that were positive for COVID.

(A)

| **Outcome** | **Non-COVID**  **(n= 188)** | **COVID**  **(n= 203)** | **OR or Between-Group Difference**  **(95% CI)** | **p** |
| --- | --- | --- | --- | --- |
| Ventilator-free days | 23.2 (7.4) | 10.0 (10.5) | 13.2 (11.4 – 15.0) | <0.01 |
| ICU-free days | 21.0 (7.7) | 7.1 (9.2) | 14.0 (12.3 – 15.7) | <0.01 |
| Hospital-free days | 15.5 (9.6) | 4.1 (7.1) | 11.4 (9.7 – 13.1) | <0.01 |
| Mortality, n (%) | 14 (7.4) | 84 (41.4) | 8.8 (4.8 – 16.2) | <0.01 |

**(B)**

| **Variable** | **aOR** | **95% CI** | ***P* value** |
| --- | --- | --- | --- |
| Early deep sedation | 2.76 | 1.26 – 6.06 | 0.01 |
| Age | 1.02 | 0.99 – 1.04 | 0.14 |
| Race | 0.90 | 0.67 – 1.20 | 0.47 |
| Total SOFA Score | 1.24 | 1.10 – 1.40 | <0.01 |
| Indication for mechanical ventilation | 1.75 | 0.59 – 5.17 | 0.31 |
